# Supercritical Carbon Dioxide Extraction of Bioactive Compounds from *Ampelopsis grossedentata* Stems: Process Optimization and Antioxidant Activity

**DOI:** 10.3390/ijms12106856

**Published:** 2011-10-14

**Authors:** Yuefei Wang, Le Ying, Da Sun, Shikang Zhang, Yuejin Zhu, Ping Xu

**Affiliations:** 1 Department of Tea Science, Zhejiang University, Hangzhou 310058, China; E-Mails: tyfwang@gmail.com (Y.W.); leying@zju.edu.cn (L.Y.); xiaodazi1987@yahoo.cn (D.S.); 2 Hangzhou Tea Research Institute, China COOP, Hangzhou 310016, China; E-Mails: zsk6510@126.com (S.Z.); zhuyuejin57@126.com (Y.Z.)

**Keywords:** *Ampelopsis grossedentata*, stems, bioactive compounds, optimization, antioxidant activity

## Abstract

Supercritical carbon dioxide (SC-CO_2_) extraction of bioactive compounds including flavonoids and phenolics from *Ampelopsis grossedentata* stems was carried out. Extraction parameters such as pressure, temperature, dynamic time and modifier, were optimized using an orthogonal array design of L_9_ (3^4^), and antioxidant activities of the extracts were evaluated by 2,2-diphenyl-1-picrylhydrazyl (DPPH) free radical scavenging assay and ferrous ion chelating (FIC) assay. The best conditions obtained for SC-CO_2_ extraction of flavonoids was 250 bar, 40 °C, 50 min, and with a modifier of methanol/ethanol (1:3, v/v), and that for phenolics extraction was 250 bar, 40 °C, 50 min, and with a modifier of methanol/ethanol (1:1, v/v). Meantime, flavonoids and phenolics were found to be mainly responsible for the DPPH scavenging activity of the extracts, but not for the chelating activity on ferrous ion according to Pearson correlation analysis. Furthermore, several unreported flavonoids such as apigenin, vitexin, luteolin, *etc*., have been detected in the extracts from *A. grossedentata* stems.

## 1. Introduction

It is well known that reactive oxygen species (ROS) are the major free radicals in human body. They may induce damage to cell structures, DNA, lipids and proteins [1], which lead to a series of diseases such as cancers, diabetes, atherosclerosis, cardiovascular disease and neurological disorders [2]. Flavonoids and phenolics are main plant-derived biocompounds and also known as natural antioxidants due to their redox properties, allowing them to act as free radical scavengers, hydrogen donors, reducing agents and metal ion chelators [3,4]. Natural antioxidants have received considerable attention due to their ability to prevent human body against oxidative stress induced by imbalance between generation and removal of reactive oxygen species and retard the progress of many chronic diseases [5].

*Ampelopsis grossedentata* (Hand-Mazz) W.T. Wang is a kind of medicinal plant, widely distributed in southern China. Its leaves and stems not only can be used as folk medicine for the treatment of hepatitis, flu, hypertension, hyperglycemic and sore throat, but also have been used as a daily drink by local people for the past few centuries [6]. Many researchers have previously focused on the bioactive compounds, such as flavonoids and phenolics, in *A. grossedentata* leaves. It has been reported that *A. grossedentata* leaves have high contents of dihydromyricetin and myricetin, and its flavonoid-rich extracts have been considered as natural antioxidants with potential application in the food industry [7,8]. Comparatively, less attention has been paid to *A. grossedentata* stems, although there are also plenty of bioactive compounds [9]. Therefore, preparing extracts enriched with bioactive compounds is essential for industrial utilization of *A. grossedentata* stems.

Conventional extraction methods, such as steam distillation and organic solvent extraction, have been used to extract bioactive compounds from plant materials for a long time. These methods usually require a long time, a large amount of solvent and high temperatures [10]. Therefore, developing alternative extraction techniques with high efficiency and moderate peculiarity is highly desirable. Supercritical carbon dioxide (SC-CO_2_) extraction has received a great deal of attention because it is usually performed at low temperatures, costing short extraction time and a small amount of solvent [11,12]. Previously, SC-CO_2_ has been used to extract flavonoids and phenolics from a wide range of plants [13–15]. Generally, addition of a small amount of a liquid polar modifier (methanol or ethanol) can significantly enhance extraction efficiency of flavonoids and phenolics [16].

The aims of this study were to employ SC-CO_2_ to extract bioactive compounds including flavonoids and phenolics from *A. grossedentata* stems, to develop an optimal extraction condition using an orthogonal array design (OAD), to evaluate antioxidant activities of the extracts by 2,2-diphenyl-1- picrylhydrazyl (DPPH) free radical scavenging assay and ferrous ion chelating (FIC) assay, respectively, and to identify the main flavonoids using high performance liquid chromatography (HPLC).

## 2. Results and Discussion

### 2.1. Optimization of SC-CO_2_ Extraction

An orthogonal array design of L_9_ (3^4^) was performed to optimize pressure, temperature, dynamic time and modifier (Table 1) at a CO_2_ flow rate of 2 L/min and a modifier flow rate of 0.5 mL/min. The obtained results (Table 2) showed that the maximum total flavonoid content (TFC) and total phenolic content (TPC) of the extracts were 4.67 mg RE/g dry material and 2.49 mg GAE/g dry material, respectively. A further orthogonal analysis is given in Table 3. The influence on TFC of extraction conditions decreased in the following order: pressure > dynamic time > temperature > modifier. Meanwhile, temperature had the dominant effect on TPC, followed by pressure, dynamic time and modifier based on the R values given in Table 3. ANOVA results (Table 4) showed that all the four parameters had a significant (*p* < 0.05) effect on both TFC and TPC of the extracts. The best conditions obtained for SC-CO_2_ extraction of flavonoids from *A. grossedentata* stems was 250 bar, 40 °C, 50 min, and with a modifier of methanol/ethanol (1:3, v/v), and that for phenolics extraction was 250 bar, 40 °C, 50 min, and with a modifier of methanol/ethanol (1:1, v/v). Moreover, whether TFC and TPC will increase under the conditions of further increasing pressure, time, concentration of ethanol, and lower temperature needs further study.

### 2.2. Effects of Various Pressures

**Figure 1:**
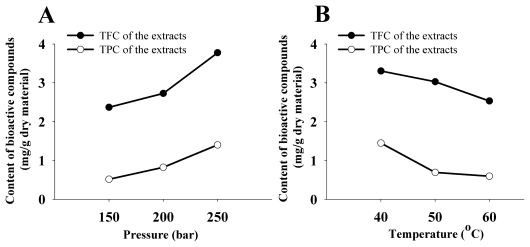

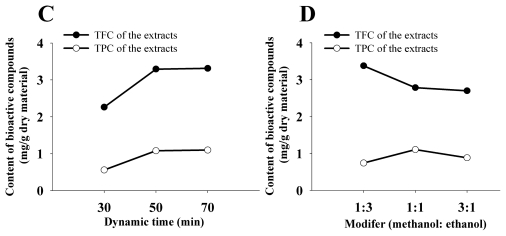
Effects of pressure (**A**), temperature (**B**), dynamic time (**C**) and modifier (**D**) on TPC and TFC of the extracts from *A. grossedentata* stems.

The effects of various pressures on TFC and TPC of the extracts from *A. grossedentata* stems are shown in [Figure 1(A)]. It can be observed that both TFC and TPC of the extracts increased as pressure varied from 150 bar to 250 bar. The same phenomenon was observed in SC-CO_2_ extraction of the bioactive flavonoid compounds from Peach Kaca [10]. This could be explained by that a higher CO_2_ density at higher pressures increases CO_2_ power to dissolve the solute and thus more bioactive compounds were extracted from *A. grossedentata* stems. While the negative effect of pressure on the extraction yields of bioactive compounds has also been reported [17–19]. Different types and contents of components in different plant materials can be responsible for that.

### 2.3. Effects of Various Temperatures

Based on the results given in Table 4, temperature was appraised as an extremely significant (*p* < 0.01) factor for the SC-CO_2_ extraction of bioactive compounds. The effects of various temperatures on TFC and TPC of the extracts are shown in [Figure 1(B)]. Both TFC and TPC of the extracts decreased as the temperature increased from 40 °C to 60 °C. Generally, temperature has a double effect on the SC-CO_2_ extractions. Higher temperature increased the vapor pressure of the solute and improved the extraction yield, while higher temperature could also reduce the density of carbon dioxide, decreased the extraction yield [20]. The results in our study demonstrated that the reduced density of carbon dioxide was the predominant rather than increased the vapor pressure of the solute.

### 2.4. Effects of Various Dynamic Times

[Figure 1(C)] shows the effects of various dynamic times on TFC and TPC of the extracts from *A. grossedentata* stems. When dynamic time varied from 30 min to 50 min, TFC and TPC of the extracts both increased obviously. While when the time increased from 50 min to 70 min, TFC and TPC enhanced only slightly. These results accorded with that of a previous research by Liu *et al.* [21]. Despite TFC (3.31 mg RE/g dry material) obtained under 70 min was higher than that under 50 min, no significant (*p* < 0.05) difference was observed between them. A similar phenomenon was observed on TPC. Thus, from the point of view of extraction efficiency, relatively shorter extraction time (less than 50 min) would be preferred in SC-CO_2_ extraction of bioactive compounds from *A. grossedentata* stems.

### 2.5. Effects of Various Modifiers

As presented in [Figure 1(D)], the highest TFC (3.38 mg RE/g dry material) of the extracts was produced under a modifier of methanol/ethanol (1:3, v/v), which was significantly (*p* < 0.05) higher than that (2.79 mg RE/g dry material) obtained under a modifier of methanol/ethanol (1:1, v/v), or that (2.70 mg RE/g dry material) obtained under a modifier of methanol/ethanol (3:1, v/v). However, the highest TPC of the extracts was obtained at a modifier of methanol/ethanol (1:3, v/v). Although the role of modifier in extraction of bioactive compounds from *A. grossedentata* stems was not as important as those of other factors, like time, pressure, and temperature in this study (Table 3), the results [Figure 1(D)] showed modifier had a selective effect on extraction of bioactive compounds due to its varied polarity.

### 2.6. DPPH Radical Scavenging Activity

**Figure 2:**
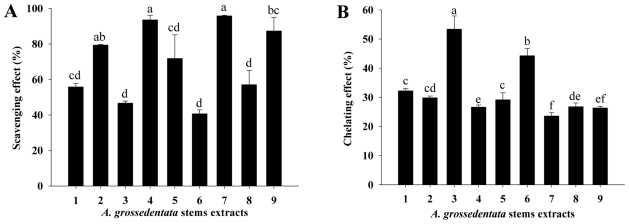
Antioxidant activities of the extracts (1–9 according to orthogonal array design (OAD)) by the 2,2-diphenyl-1-picrylhydrazyl (DPPH) radical scavenging assay (**A**) and ferrous ion chelating assay (**B**).

DPPH has been widely used to evaluate the free radical scavenging effectiveness of various antioxidant substances [22]. The mechanism of the reaction between antioxidant and DPPH depends on the structural conformation of the antioxidants. The free radical scavenging activities of flavonoids or phenolics are dependent on the presence of free OH groups, especially 3-OH [23]. The DPPH radical scavenging activities of the SC-CO_2_ extracts from *A. grossedentata* stems under different experiment conditions are given in [Figure 2(A)]. The DPPH radical scavenging effect of the extracts ranged from 40.69% to 95.87%. The extract 7 (according to OAD) exhibited the strongest DPPH radical scavenging ability, followed by the extract 4 (93.58%), and the extract 9 (87.30%). Pearson correlation analysis was used to gain a better understanding of the relationship between different antioxidant responses and the contents of total flavonoids and phenolics of the extracts. As shown in Table 5, a highly significant (*p* < 0.05) correlation coefficient (0.85) was found between DPPH scavenging activity and TPC of the extracts, and the correlation coefficient between DPPH scavenging activity and TFC of the extracts was 0.674 (*p* < 0.01). These results indicated that flavonoids and phenolics were mainly responsible for the DPPH scavenging capacity of the extracts from *A. grossedentata* stems.

### 2.7. Ferrous Ion Chelating Activity

The transition metal ion, Fe^2+^ possess the ability to move single electrons by virtue of which it can allow the formation and propagation of many radical reactions, even starting with relatively non-reactive radicals [24]. The main way to avoid ROS generation that is associated with redox active metal catalysis involves chelating of the metal ions [25]. The bioactive compounds interfered with the formation of ferrous and ferrozine complex, suggesting that they have chelating activities and captures ferrous ion before ferrozine. As shown in [Figure 2(B)], the extract 3 possessed the best ferrous ion chelating abilities (53.39%), followed by the extract 6 (44.25%), and the extract 1 (32.23%). It was surprised to observe that the extracts with relatively high ferrous ion chelating activity were not rich either in flavonoids or phenolics. Flavonoids and phenolics usually are known to act as antioxidants, both as radical scavengers and as metal chelators [26,27]. However, the extract 7, the extract 4, and the extract 9, which were abundant in bioactive compounds, showed the weakest ferrous ion chelating ability. This lack of relationship is in agreement with a previous research [28]. It was suggested that chelating ability depended on molecular structure of the flavonoids/phenolics and might be a proposal explanation for different performances on antioxidant activities of the extracts in this study [27]. As shown in Table 5, Pearson correlation coefficient between FIC and TFC of the extracts was found to be −0.740 (*p* < 0.01), and that between FIC and TPC of the extracts was −0.568 (*p* < 0.05).

### 2.8. Quantification of the Main Flavonoids

Dihydromyricetin, myricetin and several other common flavonoids, including vitexin-2″-O-rhamnoside, vitexin, rutin, quercetin-3-galactoside, quercitrin, luteolin, quercetin, apigenin, and kaempferol, were analyzed by HPLC in order to determine the main flavonoid contents of the extracts from *A. grossedentata* stems. The individual flavonoid content in the extracts is given in Table 6. The extract 7 had the highest dihydromyricetin content (2534.42 ± 195.93 μg/g dry material) and the highest total content of individual flavonoids (2550.48 ± 196.73 μg/g dry material). Previous reports have showed that dihydromyricetin and myricetin existed in *A. grossedentata* stems [9], however, its flavonoid profile have not been illustrated clearly yet. The above results demonstrated that vitexin-2″-O-rhamnoside, vitexin, rutin, quercetin-3-galactoside, quercitrin, luteolin, quercetin, apigenin, and kaempferol also existed in *A. grossedentata* stems. However, the total contents of individual flavonoids in the extracts determined by HPLC were lower than that measured by colorimetric assay. This could be explained, at least partly, by the performance of colorimetry which tended to be less accurate than that of HPLC, but it still strongly suggests that some other flavonoids in addition to those detected exist in the extracts. Hence, isolation and characterization of these flavonoids could be carried out in a further study.

## 3. Experimental Section

### 3.1. Materials

The stems of *A. grossedentata* were obtained from Sanming in Fujian Province, China. They were dried at 40 °C for 24 h and then milled into powder by an herbal pulverizer (XB-02, Xiaobao Machinery Co. Ltd., Yongkang, China). The resulting flour passed through a 20 mesh sieve and stored in a refrigerator at 4 °C until needed.

### 3.2. Chemicals

Carbon dioxide (purity 99.9%) was supplied by Zhejiang Gas Company (Hangzhou, China). Methanol and acetonitrile of HPLC grade were purchased from Tianjin Shield Company (Tianjin, China). Folin-Ciocalteu’s phenol reagent, gallic acid, rutin, 3-(2-pyridyl)-5,6-diphenyl-1,2,4-triazine- 4′,4″-disulfonic acid sodium salt (Ferrozine), 2,2-diphenyl-1-picrylhydrazyl (DPPH), and all the flavonoid standards were purchased from Sigma-Aldrich (MO, USA). All other chemicals were analytical grade and purchased from Sinopharm Chemical Reagent Co. Ltd. (Shanghai, China).

### 3.3. SC-CO_2_ Extraction

SC-CO_2_ extraction was performed on a supercritical fluid extractor Spe-ed™ SFE-2 (Applied Separation, USA). 20 g of *A. grossedentata* stems was packed into a 50 mL extraction vessel filled with defatted cotton in both ends. The flow rate of CO_2_ and modifier were maintained at 2 L/min and 0.5 mL/min, respectively. Liquid CO_2_ and modifier were pumped into the extraction vessel after desired temperature was achieved. In this study, a four-factor, three-level orthogonal array design (OAD) was chosen for optimization of SC-CO_2_ extraction of *A. grossedentata* stems. Extractions were performed at three different pressure (150, 200 and 250 bar), three different temperature (40, 50 and 60 °C), three different dynamic time (30, 50 and 70 min) and three different modifier of methanol and ethanol (1:3, 1:1 and 3:1, v/v). The extracts were collected in a glass vial at room temperature and atmospheric pressure. The modifier was removed completely by a vacuum rotary evaporator (R205B, Shensheng Co. Ltd., Shanghai, China) at 40 °C (water-bath temperature). The dry extracts were adjusted to 50 mL with absolute ethanol as samples for further analysis.

### 3.4. Determination of TFC of the Extracts

TFC of the extracts was measured using the method of Kim *et al*. [29] with minor modifications. In brief, 0.5 mL of sample was added to a 10 mL volumetric flask containing 5 mL absolute ethanol. Then 0.3 mL of 5% (w/v) NaNO_2_ was added to the flask. After 5 min, 0.3 mL of 10% (w/v) Al (NO_3_)_3_ was added. At 6 min, 4 mL of NaOH (1 M) was added to the mixture and adjusted to 10 mL with absolute ethanol. The mixture was thoroughly mixed and the absorbance was measured at 510 nm (HP 8453 UV-Vis spectrophotometer, Hewlett Packard, Palo Alto, CA, USA). A calibration curve was obtained with rutin. TFC of the extracts was expressed as rutin equivalents (mg RE/g dry material).

### 3.5. Determination of TPC of the Extracts

TPC of the extracts was determined using the Folin-Ciocalteu method by Meda *et al*. [30] with a slight modification. Briefly, 1 mL of sample was transferred into a 10 mL volumetric flask and mixed with 6 mL of distilled water. To each sample, 0.5 mL of 50% (v/v) Folin-Ciocalteu reagent was added and mixed. After 5 min, 1 mL of 5% (w/v) Na_2_CO_3_ was added to the mixture and adjusted to 10 mL with distilled water. After standing for 60 min at room temperature, the absorbance was measured at 760 nm. Gallic acid was used for constructing the standard curve. TPC of the extracts was expressed as gallic acid equivalents (mg GAE/g dry material).

### 3.6. DPPH Free Radical Scavenging Activity

The DPPH free radical scavenging activity of the extracts was determined according to the method of Liu *et al*. [31] with a slight modification. 200 μL of sample was added to 7.8 mL of ethanolic DPPH solution (60 μM). After vortexing the reaction mixture for 1 min, the tubes were kept in dark for 30 min and the absorbance (*A*_1_) was measured at 517 nm. A control containing the same amount of absolute ethanol and DPPH radical was prepared and measured at the same wavelength (*A*_0_). The DPPH radical scavenging effect (%) was calculated as the following equation:

$$\text{DPPH scavenging effect }(\%)=(1-A_{1}/A_{0})\times 100$$

### 3.7. Chelating Effect on Ferrous Ion

The chelating effect of the extracts on ferrous ion was assayed according to Wang *et al*. [32] with a few modifications. 1 mL of the sample was mixed with 1 mL of FeSO_4_ (0.1 mM) for 30 s, then 1 mL ferrozine (0.25 mM) was added and the mixture was kept for 10 min at room temperature. The absorbance of the mixture was determined at 562 nm (*A*_1_). A reagent control was measured by the same way (*A*_0_) and the ability of sample for the ferrous ion was calculated as the following equation:

$$\text{Chelating effect }(\%)=(1-A_{1}/A_{0})\times 100$$

### 3.8. High performance liquid chromatography (HPLC) analysis

**Figure 3:**
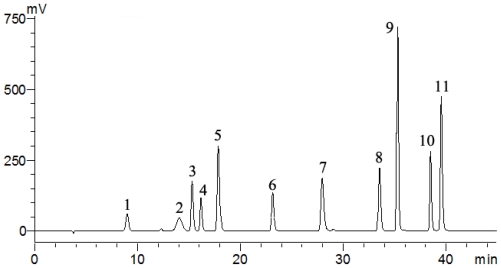
The high performance liquid chromatography (HPLC) chromatogram of standard mixture solution. Peaks: 1: Dihydromyricetin; 2: Vitexin-2″-O-rhamnoside; 3: Vitexin; 4: Rutin; 5: Quercetin-3-galactoside; 6: Quercitrin; 7: Myricetin; 8: Luteolin; 9: Quercetin; 10: Apigenin; 11: Kaempferol.

The main flavonoid components of the extracts were analyzed using a high performance liquid chromatography (HPLC) method. Flavonoid standards including dihydromyricetin, vitexin-2″-O-rhamnoside, vitexin, rutin, quercetin-3-galactoside, quercitrin, myricetin, luteolin, quercetin, apigenin, and kaempferol were prepared at 1 mg/mL in absolute ethanol. The HPLC analysis was performed with a Shimadzu SPD-20A ultraviolet detector, a SIL-20AC automatic sample injector (Shimadzu Inc. Ltd., Tokyo, Japan) and equipped with an Agilent TC-C18 reversed-phase column (4.6 mm × 150 mm × 5 μm). The temperature of the column during analysis was maintained at 35 °C. The mobile phase consisted of solvent A (3% acetonitrile + 0.5% acetic acid) and solvent B (50% acetonitrile + 0.5% acetic acid) with the elution profile as follows: 0–24 min, 27.5–45% B (linear gradient, v/v); 24–29 min, 40–80% B (linear gradient); 29–37 min, 80% B; 37–45 min, 27.5% B (equilibration). The flow rate was kept constant at 1.0 mL/min and the peaks were identified using UV absorbance at 360 nm. The injection volume was 10 μL each time. The HPLC chromatogram of standard mixture solution was presented in Figure 3.

### 3.9. Statistical Analysis

All the experiments were carried out in triplicate. The results were expressed as means ± SD and evaluated by analysis of variance (ANOVA) followed by Turkey’s studentized range test carried out on the SAS system for windows (Version 9.1, SAS Institute Inc., Cary, NC, USA). Pearson’s correlation tests were performed on SPSS for Windows (Version 16.0, Chicago, IL, USA).

## 4. Conclusions

Based on the results obtained, the best conditions obtained for SC-CO_2_ extraction of flavonoids from *A. grossedentata* stems was 250 bar, 40 °C, 50 min and with a modifier of methanol/ethanol (1:3, v/v), and those for phenolics extraction was 250 bar, 40 °C, 50 min and with a modifier of methanol/ethanol (1:1, v/v). Meanwhile, flavonoids and phenolics were found to be mainly responsible for the DPPH scavenging activity of the extracts, but not for the chelating activity on ferrous ion according to Pearson correlation analysis. Furthermore, several unreported flavonoids such as apigenin, vitexin, luteolin, *etc*, have been detected in the extracts from *A. grossedentata* stems. These results indicate that SC-CO_2_ could be a promising alternative for preparation of extracts enriched with bioactive compounds from *A. grossedentata* stems. These extracts have effective antioxidant capacity and could act as different kinds of natural antioxidant agents. While other bioactivity, like antiviral and bactericide capability, of the extracts obtained from *A. grossedentata* stems, and using other “natural solvents”, like aqueous ethanol mixtures, to replace methanol as modifier of SC-CO_2_ extraction need to be investigated in future research.

## Figures and Tables

**Table 1:** The factors and levels of the orthogonal array design.

Factors	Levels		
	1	2	3
Pressure (bar)	150	200	250
Temperature (°C)	40	50	60
Dynamic time (min)	30	50	70
Modifier (methanol: ethanol, v/v)	1:3	1:1	3:1

**Table 2:** Results obtained under the experimental conditions using L_9_ (3^4^) orthogonal array design.

Trial	Pressure (A)	Temperature (B)	Dynamic Time (C)	Modifier (D)	TFC (mg RE/g Dry Material) a	TPC (mg GAE/g Dry Material) a
1	1	1	1	1	2.56 ± 0.03 cd	0.53 ± 0.02 c
2	1	2	2	2	2.62 ± 0.17 cd	0.73 ± 0.04 c
3	1	3	3	3	2.20 ± 0.86 cd	0.39 ± 0.03 c
4	2	1	2	3	3.40 ± 0.33 abc	1.55 ± 0.41 b
5	2	2	3	1	3.60 ± 0.19 abc	0.53 ± 0.17 c
6	2	3	1	2	1.60 ± 0.32 d	0.35 ± 0.04 c
7	3	1	3	2	4.67 ± 0.36 a	2.49 ± 0.10 a
8	3	2	1	3	2.97 ± 0.20 bcd	0.57 ± 0.03 c
9	3	3	2	1	4.24 ± 0.01 ab	0.84 ± 0.11 c

a Values in the same column followed by different letters are significantly different (*p* < 0.05).

Total flavonoid content (TFC); total phenolic content (TPC)

**Table 3:** Analysis of L_9_ (3^4^) orthogonal array design results.

	TFC (mg RE/g Dry Material)				TPC (mg GAE/g Dry Material)			
	Pressure (A)	Temperature (B)	Dynamic Time (C)	Modifier (D)	Pressure (A)	Temperature (B)	Dynamic Time (C)	Modifier (D)
*K*_1_	7.37 a	10.63	7.12	10.39	1.65	4.57	1.45	1.90
*K*_2_	8.60	9.18	10.26	8.88	2.44	1.84	3.13	3.57
*K*_3_	11.87	8.03	10.46	8.57	3.90	1.58	3.41	2.51
*k*_1_	2.46 b	3.54	2.37	3.46	0.55	1.52	0.48	0.63
*k*_2_	2.87	3.06	3.42	2.96	0.81	0.61	1.04	1.19
*k*_3_	3.96	2.68	3.49	2.86	1.30	0.53	1.14	0.84
*R*	4.50 c	2.59	3.34	1.81	2.25	2.98	1.96	1.67

a *K*_i_^A^ = ∑ the amount of target compounds at *A*_i_;

b *k*_i_^A^ = *K*_i_^A^/3;

c *R*_i_^A^ = max {*k*_i_^A^} − min{*k*_i_^A^}.

**Table 4:** ANOVA analysis of four parameters for supercritical carbon dioxide **(**SC-CO_2_) extraction.

Source	Sum of Squares	DF	Mean Square	*F*-Value	*p*-Value
TFC					
Corrected Model a	21.341	8	2.667	17.180	0.000
Pressure	9.638	2	4.819	31.040	0.000
Temperature	2.745	2	1.373	8.840	0.002
Dynamic time	6.500	2	3.250	20.930	0.000
Modifier	2.458	2	1.229	7.910	0.003
TPC					
Corrected Model b	9.911	8	1.239	19.700	0.000
Pressure	3.635	2	1.817	28.900	0.000
Temperature	3.945	2	1.972	31.370	0.000
Dynamic time	1.716	2	0.858	13.64	0.000
Modifier	0.616	2	0.308	4.890	0.020

a *R*^2^ = 0.884;

b *R*^2^ = 0.898.

**Table 5:** Pearson correlation coefficient analysis.

	TFC	TPC	DPPH	FIC
TFC	1.000	0.710 **	0.674 **	−0.740 **
TPC		1.000	0.850 *	−0.568 *
DPPH			1.000	−0.677 **

** Correlation is significant at *p* < 0.01;

* Correlation is significant at *p* < 0.05.

**Table 6:** Quantification of the main flavonoids from *A. grossdentata* stems (μg/g dry material) a.

	Dihydromyricetin	Vitexin-2″-O-rhamnoside	Vitexin	Rutin	Quercetin-3-galactoside	Quercitrin	Myricetin	Luteolin	Quercetin	Apigenin	Kaempferol	Total Content
1 b	210.01 ± 8.33 de	-	1.12 ± 0.01 c	-	-	-	0.43 ± 0.11 c	-	-	0.16 ± 0.02 b	0.59 ± 0.02 c	212.31 ± 8.48 de
2	386.23 ± 31.04 d	-	0.23 ± 0.07 e	3.03 ± 0.31 ab	-	-	0.68 ± 0.06 c	0.12 ± 0.00 d	0.05 ± 0.00 ab	0.28 ± 0.02 b	0.92 ± 0.01 b	391.53 ± 31.49 d
3	43.88±16.55 e	-	0.27 ± 0.03 de	2.46 ± 0.13 bc	-	-	-	0.12 ± 0.00 d	0.06 ± 0.00 ab	0.28 ± 0.01 b	1.11 ± 0.01 a	48.16 ± 16.43 e
4	1476.48 ± 38.08 b	0.34 ± 0.14 abc	0.45 ± 0.07 ed	3.83 ± 0.11 a	0.18 ± 0.06 b	0.74 ± 0.06 a	2.83 ± 0.34 b	0.45 ± 0.16 b	0.05 ± 0.00 ab	1.46 ± 0.14 a	0.86 ± 0.03 b	1488.66 ± 39.48 b
5	436.20 ± 3.68 d	0.32 ± 0.18 bc	0.56 ± 0.18 d	3.70 ± 0.86 a	-	-	-	0.17 ± 0.02 cd	0.05 ± 0.01 ab	1.63 ± 0.12 a	-	442.62 ± 2.29 d
6	34.41 ± 5.49 e	-	-	1.70 ± 0.05 c	-	-	-	0.15 ± 0.04 cd	0.04 ± 0.01 b	1.54 ± 0.06 a	-	37.84 ± 5.42 e
7	2534.42 ± 195.93 a	0.51 ± 0.00 ab	2.31 ± 0.00 b	-	0.39 ± 0.03 a	0.39 ± 0.03 b	8.68 ± 0.35 a	1.15 ± 0.06 a	0.04 ± 0.00 b	1.07 ± 0.39 a	0.21 ± 0.08 d	2550.48 ± 196.73 a
8	412.01 ± 27.92 d	0.47 ± 0.13 ab	2.07 ± 0.00 b	-	-	-	0.93 ± 0.10 c	0.22 ± 0.07 bcd	0.05 ± 0.01 ab	0.27 ± 0.02 b	0.90 ± 0.02 b	416.94 ± 28.23 d
9	808.13 ± 40.73 c	0.69 ± 0.03 a	3.16 ± 0.14 a	-	-	-	1.16 ± 0.24 c	0.41 ± 0.05 bc	0.07 ± 0.00 a	0.35 ± 0.03 b	0.95 ± 0.08 ab	814.92 ± 40.22 c

a Values in the same column followed by different letters are significantly different (*p* < 0.05);

b *A. grossedentata* stems extracts (1–9 according to OAD).
